# NIR in, far-red out: developing a two-photon fluorescent probe for tracking nitric oxide in deep tissue†
†Electronic supplementary information (ESI) available: Experimental detail, synthesis and characterization, spectroscopic properties of **NRNO**, cytotoxicity assay, TPM images of **NRNO** stained liver tissues under z-scan model, NMR and HR-MS data. See DOI: 10.1039/c6sc01313a


**DOI:** 10.1039/c6sc01313a

**Published:** 2016-04-26

**Authors:** Zhiqiang Mao, Wenqi Feng, Zhen Li, Lingyu Zeng, Weijie Lv, Zhihong Liu

**Affiliations:** a Key Laboratory of Analytical Chemistry for Biology and Medicine (Ministry of Education) , College of Chemistry and Molecular Sciences , Wuhan University , Wuhan 430072 , China . Email: zhhliu@whu.edu.cn

## Abstract

As a pivotal signalling molecule involved in various physiological and pathological processes, nitric oxide (NO) has motivated increasing interest in the last few decades. Although a considerable number of fluorescent probes have been developed for NO imaging, the *in situ* tracking of this gas molecule in biological events remains a big challenge, mainly because of the relatively short excitation and/or emission wavelengths, which are subject to background interference and lowered collection efficiency in deep-tissue imaging. Herein, we report a far-red emissive (650 nm) two-photon (TP) excitable **NRNO** probe, using Nile Red as the TP fluorophore, for NO detection and imaging both *in vitro* and *in vivo*. The **NRNO** probe shows a fast (within 180 s) and specific fluorescence response toward NO with a limit of detection (LOD) as low as 46 nM. The excellent properties of **NRNO** enable it to sensitively detect both exogenously and endogenously generated NO in living cells. The “NIR in” and “far-red out” lights lead to improved penetrating ability, thus endowing the probe with high resolution for the illumination of deep tissues. It is therefore able to visualize the NO generation in a lipopolysaccharide (LPS)-mediated inflammation process for the first time. Our results demonstrate that **NRNO** could be a practical tool for studying the NO-related biological events. Moreover, this study also suggests the possibility of using Nile Red and its derivatives to develop far-red emissive TP probes, which is an important, yet undeveloped area.

## Abstract 2

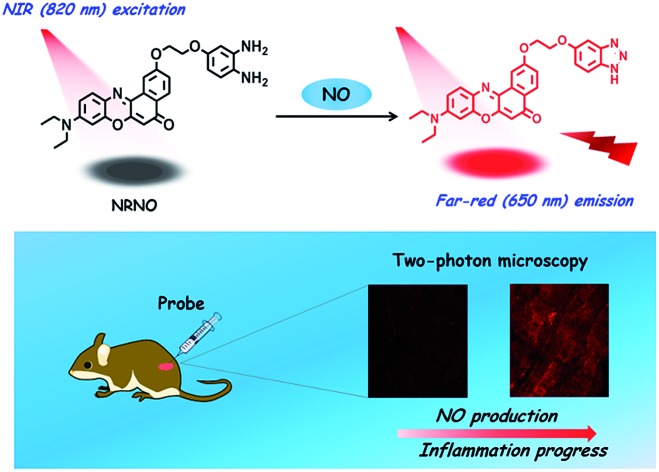
The first far-red emissive, two-photon fluorescent probe for nitric oxide was rationally designed and applied in detecting nitric oxide in both cells and tissues.

## Introduction

The *in situ* tracking of active species in live organisms is essential for understanding their biological roles. It has been the focal point of research for years and poses considerable challenges to current detection techniques. Nitric oxide (NO) has long been recognized as a pivotal signalling molecule involved in various physiological and pathological processes, such as the cardiovascular, immune, and nervous systems.^1^ As a Janus molecule in the human body, nitric oxide has triggered substantial interest in both the chemistry and biology communities.^2^ Accumulating evidence suggests that the malfunctioning of NO homeostasis is associated with numerous diseases, such as Alzheimer's disease, ischemia-reperfusion injury, cancers, and so forth.^3^ Studies have also demonstrated that NO can both promote and suppress tumour progression and metastasis, depending on the concentration and duration of NO exposure.^4^ Therefore, the concentration and diffusion dynamics of this gas at the cellular and tissue levels is an ongoing focus of investigations.

Small-molecule based fluorescence imaging has shown promise and has become an indispensable tool for studying biological specimens in life sciences, owing to its high sensitivity, specificity, real-time detection and non-invasive features, as well as technical simplicity. To date, two main types of fluorescent probes, *i.e.*, *o*-phenylenediamine and transition metal based organic dyes and complexes, have been developed to detect NO in aqueous media and cells.^5^ Despite the appreciable progress made in this area, very few probes are competent for practical applications, mainly because of their photophysical limitations; *i.e.*, their relatively short excitation/emission wavelengths restrict the illumination of deep issues with minimized background.^6^

It has been established that with the use of two-photon probes, which simultaneously absorb two NIR photons (700–900 nm), comes the ability to acquire deeper penetration, reduce background fluorescence and provide higher 3D temporal-spatial resolution than one-photon probes.^7^ During the last few years, several two-photon fluorescent probes for NO imaging in cells and tissues have been exploited by some groups and have contributed considerably to the understanding of the biological roles of NO.^8^ However, these TP probes circumvent only half of the above problem (by locating the excitation in the NIR region), and the deficiency with emission wavelength still exists; *i.e.*, all these TP probes emit in the green region or shorter wavelength (<550 nm). The short emission wavelength somewhat impairs the aforementioned advantages of TP probes in bio-imaging, since some intrinsic biomolecules (such as NADH, riboflavin, and folic acid *etc.*) also can be two-photon excited and lead to background fluorescence in the blue to green region.^9^ Meanwhile, the <550 nm emission of the TP probes can be absorbed by biomolecules like hemoglobin (Hb) and oxyhemoglobin (HbO_2_), which decrease the collection efficiency of the fluorescence signal in deep imaging.^10^ Therefore, it would be more significant to develop TP probes with the emission in the far-red to NIR region, which is much less absorbed by biomolecules and penetrates deeper into tissues, so as to further minimize the interference from background fluorescence and increase the collection efficiency.^11^ Until now, due to the limitations of the fluorophores, which should possess both a large TP cross section and long-wavelength emission, far-red emissive^12^ TP fluorescent probes have rarely been reported.

In this work, we designed and synthesized a far-red emissive two-photon fluorescent probe for the detection of nitric oxide in living systems. The **NRNO** probe exhibited a prominent fluorescence enhancement upon reaction with NO in the far-red region in a short time, with excellent selectivity and high sensitivity. Owing to its photophysical merits, the probe was successfully used to measure both exogenous and endogenous NO in living cells. It was also able to vividly image NO in liver tissue slices of mice at a depth up to 170 μm with high resolution, and thus, it was capable of monitoring the production of NO in the LPS-mediated inflammation process. To the best of our knowledge, this is the first far-red emissive two-photon probe for nitric oxide that realizes the *in situ* tracking of NO in the pathological event.

## Results and discussion

### Design and synthesis of **NRNO**

**Scheme 1:**
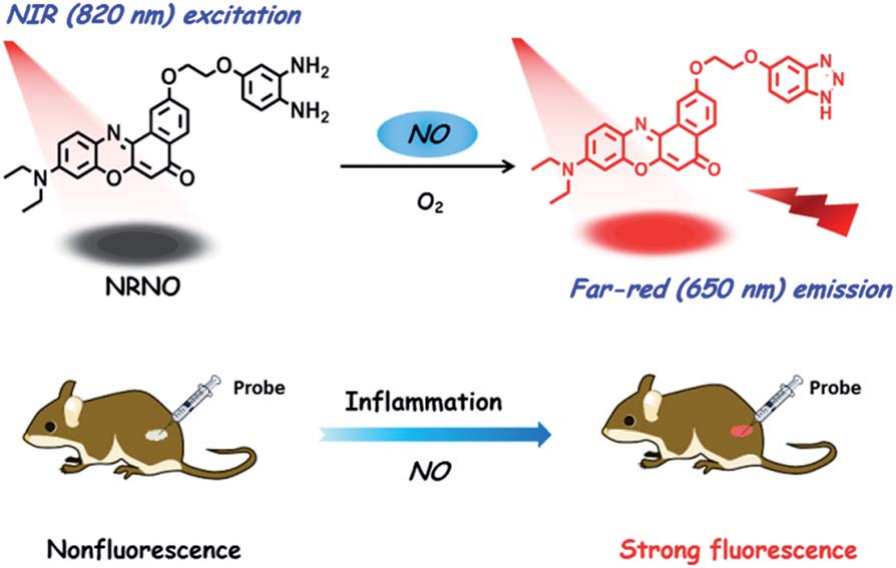
Design of the far-red emissive two-photon fluorescent probe, **NRNO**, for nitric oxide (NO) and its application in the detection of nitric oxide in inflamed tissues.

Despite the advancement of TP microscopy in the last decade, TP probes with far-red/NIR emission are rarely reported. The main reason is the lack of long-wavelength emissive TP fluorophores with sufficient two-photon action cross sections. In our design of **NRNO**, we chose Nile Red as the fluorescence reporting unit for its attractive photophysical properties, including the long emission wavelength (>600 nm), large extinction coefficient and good photostability.^13^ Owing to the easily polarized electronic structure and large conjugated π system,^14^ we speculated that Nile Red might be an excellent platform for developing red-emissive two-photon probes. *O*-Phenylenediamine was selected as the receptor of nitric oxide for its specific response and high sensitivity, and it is a common photoinduced electron transfer (PET) quencher for most fluorophores.^15^ On the other hand, it is well-established that the PET quenching efficiency is distance dependent; *i.e.*, it decreases with increasing distance between the electron donor and electron acceptor.^16^ To guarantee the efficiency of fluorescence quenching, we covalently linked the fluorescence reporting unit and *o*-phenylenediamine unit with a short oxygen-containing, four-membered chain (–OCH_2_CH_2_O–, Scheme 1). It is envisioned that the **NRNO** probe shows weak fluorescence due to the PET effect of the *o*-phenylenediamine unit. When **NRNO** reacts with nitric oxide under aerobic conditions, the *o*-phenylenediamine transforms into triazole and the product should emit intensely in the far-red region because of the inhibition of the PET effect (Scheme 1). The compound NRNO was synthesized and characterized by ^1^H NMR, ^13^C NMR and HR-MS spectra (Fig. S1 and S8–S16, ESI†).

### Spectroscopic properties of **NRNO** and its response toward NO

**Fig. 1:**
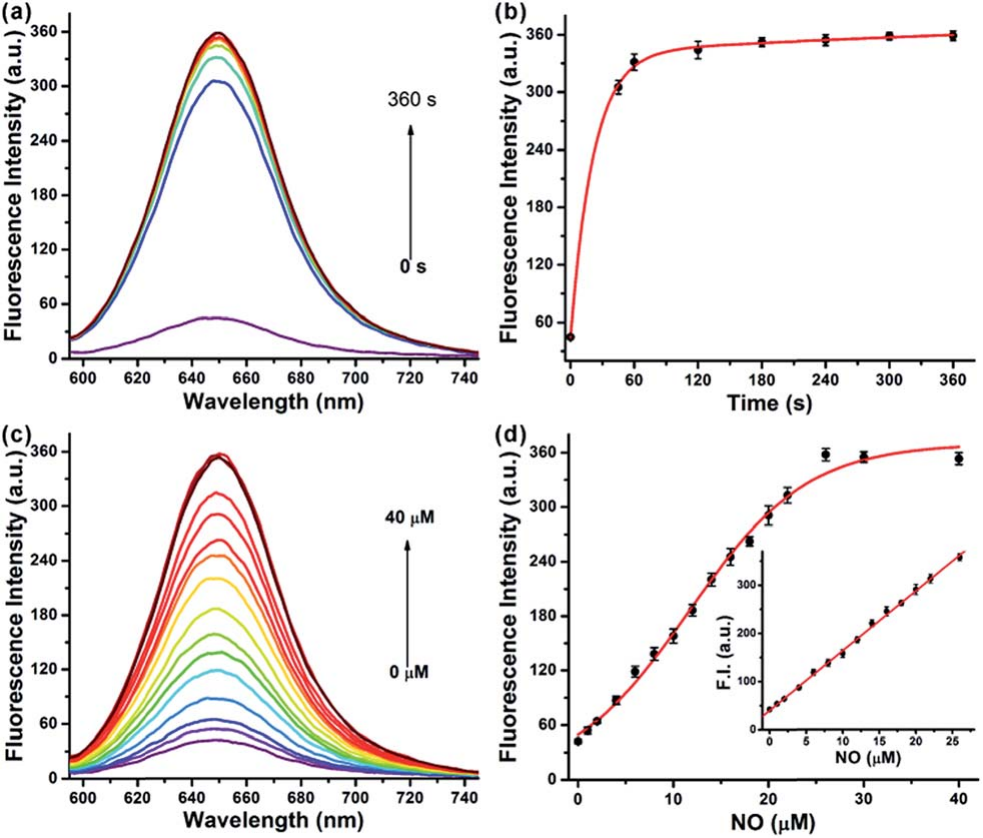
(a) Fluorescence spectra for the reaction of 5.0 μM **NRNO** with excess NO (30 μM) with time. (b) Plot of fluorescence intensity *vs.* time (0–360 s) for the reaction of 5.0 μM **NRNO** with 30 μM NO. (c) Fluorescence spectra of 5.0 μM **NRNO** with NO (0–40 μM) after reacting for 180 s. (d) Fluorescence intensity of **NRNO** as a function of NO concentration (0–40 μM). Inset: the linear relationship between fluorescence intensity and NO concentration within 1.0–26 μM. The excitation wavelength was 585 nm, and the fluorescence intensity was measured at 650 nm. Measurements were done in 10 mM PBS (pH = 7.4, containing 10% DMF).

We first evaluated the fluorescence properties of **NRNO** (5.0 μM) in 10 mM PBS (pH = 7.4, containing 10% DMF as co-solvent). The **NRNO** probe showed absorption and emission maxima at 583 nm (*ε* = 4.00 × 10^4^ M^–1^ cm^–1^) and 648 nm, respectively. The emission of **NRNO** in the far-red region is longer than that of previous NO probes, which can be beneficial to imaging in biological samples. The very weak fluorescence at 648 nm (with *Φ* = 0.03) is ascribed to the effective PET process. Upon reaction with excess NO, remarkable fluorescence enhancement was observed as a result of the blocking of the PET process, and the product showed an intense fluorescence emission band centered at 650 nm (*Φ* = 0.42) under the excitation of 585 nm (*ε* = 3.96 × 10^4^ M^–1^ cm^–1^) (Table S1 and Fig. S2, ESI†). The significant reaction-induced enhancement of the fluorescence quantum yield suggests that **NRNO** is suitable for use in aqueous media and rationalizes our probe design as well. We then conducted fluorescence titration of 5.0 μM of probe with external standard NO for *in vitro* detection. Firstly, we examined the kinetics of the reaction between the probe and target. The addition of 6.0 eq. of NO to the probe solution at 37 °C led to an immediate 7.4-fold increase in fluorescence intensity in 60 seconds. The signal intensity of the reaction reached a plateau at only 180 s with a fluorescence enhancement factor (*F*/*F*_0_) of *ca.* 8.4-fold (Fig. 1a and b). The fast response of the **NRNO** probe towards NO is very favourable for the real-time detection of NO in living systems. The fluorescence intensity of the reaction at 650 nm also increased linearly with the concentration of NO in the range of 1.0–26.0 μM (*R*^2^ = 0.999) (Fig. 1c and d). The limit of detection (LOD) for nitric oxide as calculated by 3*σ*/*m* criteria (where *m* is the slope for the range of the linearity used and *σ* is the standard deviation of the blank, *n* = 11) was 46 nM, which represents a high enough sensitivity for the detection of physiological NO level (nanomole to micromole).^17^

### Selectivity and pH independence of **NRNO**

The selectivity of the **NRNO** probe toward NO over other biologically relevant species was investigated. We examined the fluorescence response of **NRNO** to NO and other possible interfering molecules and ions, which include 50 μM metal ions (Ca^2+^, Mg^2+^, Zn^2+^, Mn^2+^, Fe^2+^), 1.0 mM biothiols (GSH, Cys, Hcy), 30 μM reactive oxygen species (˙OH, ^1^O_2_, H_2_O_2_, ClO^–^, O_2_^–^) and reactive nitrogen species (NO_2_^–^, ONOO^–^). As shown in Fig. S3,† none of the above interfering species caused obvious fluorescence intensity enhancement of **NRNO**, and the response of **NRNO** toward NO was not affected by the coexistence of these species. The effect of pH on the **NRNO** probe in the absence and presence of NO were also studied. As shown in Fig. S4,† both the probe itself and the reaction product were insensitive to pH in the physiological range of 6.0–8.5. These properties ensure the reliability of using **NRNO** to detect NO in biological environments.

### TP fluorescence properties of **NRNO**

The two-photon action cross sections (*Φδ*) of a probe before and after reacting with the target are crucial parameters for two-photon microscopy, which generally determine the signal enhancement factor and the signal-to-background ratio under the microscope. The two-photon action cross sections of **NRNO** in the absence and presence of NO were measured in the 10 mM PBS (pH = 7.4, containing 10% DMF as co-solvent). Similar to the above mentioned very low quantum yield, the **NRNO** probe itself possessed an extremely low TP action cross section, which was non-detectable under our experimental conditions, while the reaction product of **NRNO** and NO showed the maximal TP action cross section value of 38 GM (1 GM = 10^–50^ cm^4^ s per photon per molecule) at 820 nm (Table S1 and Fig. S5, ESI†). This result indicates that the probe has a distinct change in TP fluorescence properties before and after reacting with the target, which implies that **NRNO** is suitable for tracking NO under TP excitation.

### TP imaging of exogenous and endogenous NO in living cells

**Fig. 2:**
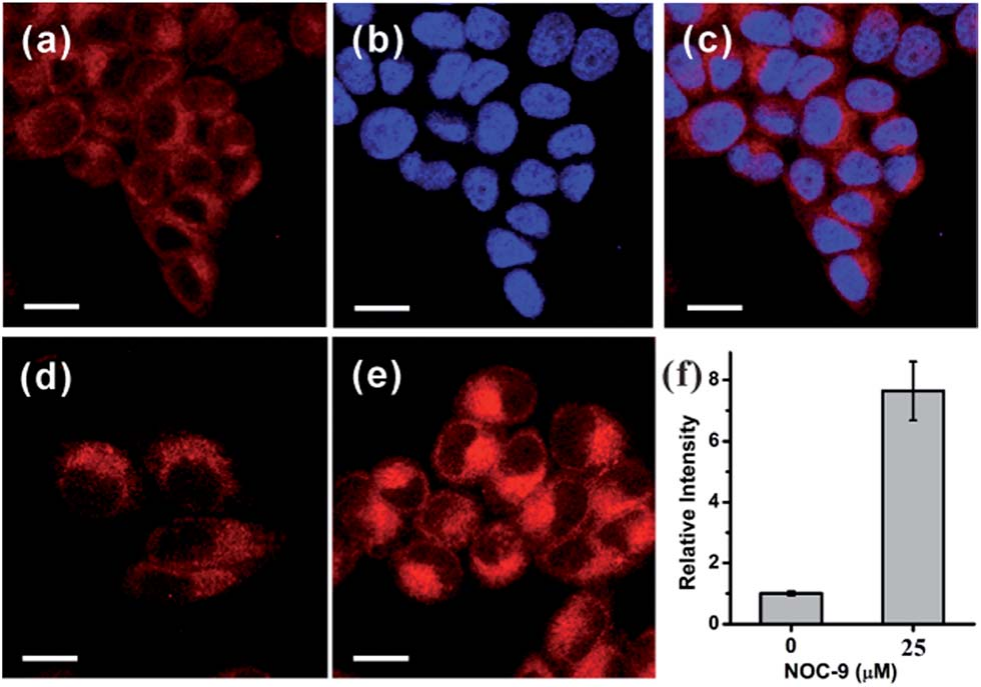
(a) TP and (b) one-photon images of HeLa cells co-stained with 5.0 μM **NRNO** and 1.0 μM Hoechst 33258 for 30 min. (c) Merged images of (a) and (b). (d) TP image of 5.0 μM **NRNO**-loaded HeLa cells. (e) TP image of HeLa cells loaded with 5.0 μM **NRNO** and then incubated with 25 μM NOC-9 for 30 min. (f) Relative TP fluorescence intensity in (d) and (e). The excitation wavelength for one-photon and two-photon imaging were 405 nm and 820 nm, respectively, and the emissions were collected at 420–500 nm for Hoechst 33258 and 620–700 nm, respectively for **NRNO**. Scale bars: 10 μm.

Based on the promising photophysical properties of **NRNO** and its rapid and selective response to NO, we proceeded to utilize **NRNO** to track NO level in living cells. The cytotoxicity of **NRNO** was first evaluated by MTT assays with HeLa cells. HeLa cells were incubated with various concentrations (5–40 μM) of **NRNO**, and the results showed that the cells had a high viability (>90%) even when treated with 25 μM **NRNO** after 24 h, demonstrating the low cytotoxicity of the probe (Fig. S6, ESI†). To clarify the intracellular localization of the probe, HeLa cells were co-incubated with **NRNO** and a well-known nucleus staining dye, Hoechst 33258.^18^ The **NRNO** and Hoechst 33258 co-stained HeLa cells exhibited red fluorescence in the whole cytoplasm when excited with TP mode at 820 nm (Fig. 2a), while the cells showed blue fluorescence in the nucleus region when excited with one-photon mode at 405 nm (Fig. 2b). The merged images (Fig. 2c) further prove that the **NRNO** probe can be easily loaded into cytoplasm without any leakage and the cells are viable throughout the imaging experiments. To test the ability of the probe to respond to exogenous NO in living cells, HeLa cells were incubated with 5.0 μM **NRNO** followed by incubation with 25 μM NOC-9, a recognized NO donor.^19^ As demonstrated in Fig. 2d and e, the red fluorescence in the cytoplasm region was significantly enhanced (7.4-fold, as quantitatively calculated and presented in Fig. 2f) after incubating with NOC-9 for 30 min. These results confirm the capability of NRNO to detect exogenous NO in living cells.

**Fig. 3:**
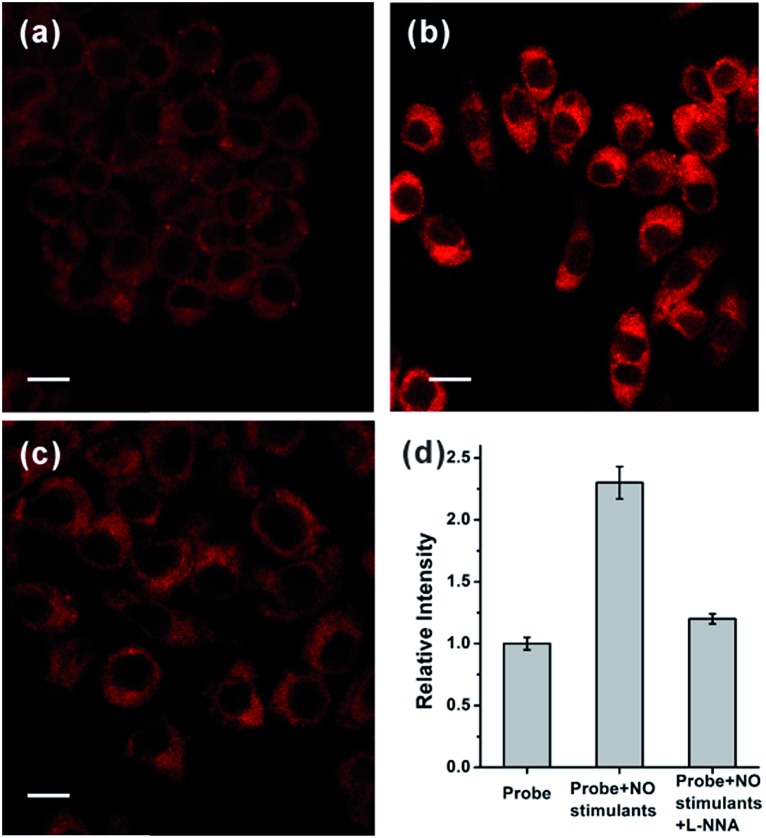
(a) TP image of RAW 264.7 cells incubated with 5.0 μM **NRNO** for 30 min. (b) TP image of RAW 264.7 cells pre-treated with NO stimulants (20 μg mL^–1^ LPS, 200 U mL^–1^ IFN-γ and 0.5 mg mL^–1^l-arginine) for 14 hours before incubation with 5.0 μM **NRNO** for 30 min. (c) TP image of RAW 264.7 cells pre-treated with NO stimulants (20 μg mL^–1^ LPS, 200 U mL^–1^ IFN-γ, 0.5 mg mL^–1^l-arginine) and 10 μM l-NNA for 14 hours, before incubation with 5.0 μM **NRNO** for 30 min. (d) Relative TP fluorescence intensities in (a–c). The excitation wavelength for TP imaging was 820 nm and the emission was collected at 620–700 nm. Scale bar: 10 μm.

Motivated by the above results, we further tried to use the probe to monitor the change in endogenously generated nitric oxide in living cells under a two-photon microscope. There are previous reports on the NO production in RAW 264.7 cells stimulated by bacterial LPS and IFN-γ.^20^ We therefore selected this cell model for the endogenous NO observations. As shown in Fig. 3a, RAW 264.7 cells treated with only **NRNO** showed moderate red fluorescence, which could have originated from the probe itself and the intrinsic basal NO. When the cells were pre-treated with 20 μg mL^–1^ LPS, 200 U mL^–1^ IFN-γ and 0.5 mg mL^–1^l-arginine (the substrate for nitric oxide synthase) before the loading of the probe, a remarkable enhancement of fluorescence intensity was observed (Fig. 3b). To further prove that the enhanced fluorescence was induced by nitric oxide, we performed another control set where the cells were pre-treated with LPS, IFN-γ and l-arginine, together with 10 μM l-N^G^-nitroarginine (l-NNA), which is a known inducible NO synthase (iNOS) inhibitor.^21^ In this case, the fluorescence intensity was almost attenuated to the basal level (Fig. 3c and d). These results have unambiguously confirmed that the **NRNO** probe is capable of monitoring the fluctuation of endogenously generated NO in living cells.

### TP tissue imaging of NO generation in an inflamed mouse model

**Fig. 4:**
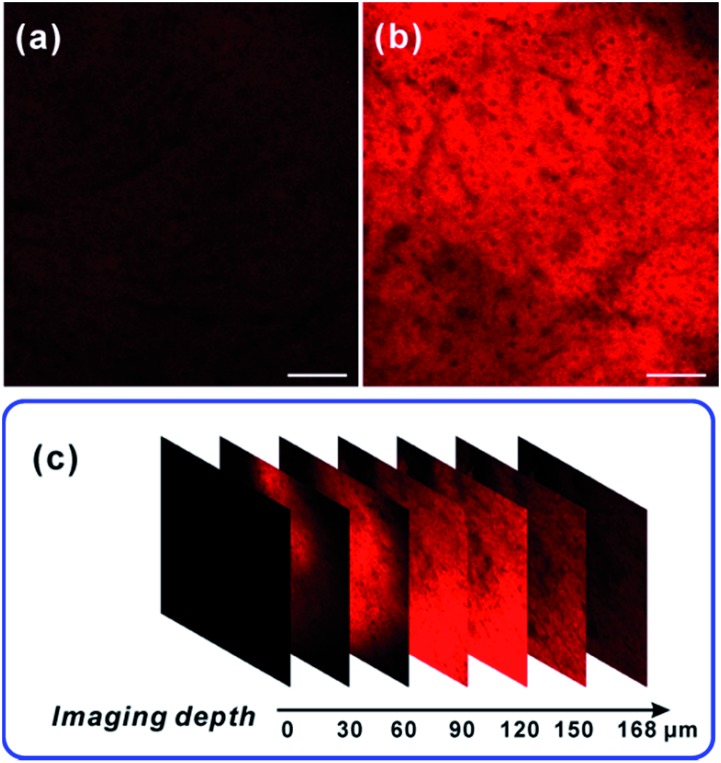
TP images of mouse liver slices at 90 μm depth, stained with 10 μM of **NRNO** for 1.5 h (a) and then with 60 μM NO for another 1 h (b). (c) The confocal z-scan TP imaging sections at different depths for 0, 30, 60, 90, 120, 150, 168 μm. The TP excited fluorescence was collected at 620–700 nm upon excitation at 820 nm with a femtosecond pulse laser. Scale bar: 100 μm.

After verifying the ability of **NRNO** to track both exogenous and endogenous NO in living cells, we attempted to show the *in vivo* applicability of this far-red emissive TP probe. Firstly, we checked the possibility of using the probe to stain and respond to NO in mouse liver tissues. To this end, TP imaging of nitric oxide in tissue slices of mouse liver was carried out. As shown in Fig. 4, a similar trend to that in living cells was obtained, *i.e.*, the **NRNO** stained tissue displayed quite weak fluorescence (Fig. 4a), whereas, the tissue treated with both **NRNO** and excess NO exhibited much brighter fluorescence (Fig. 4b). The significant change in TP fluorescence intensity proved that the probe is suitable for the detection of NO in deep tissues. By using the z-scan mode of the TP microscope, the fluorescence intensities at different depths of mouse liver tissue were recorded (Fig. 4c and Fig. S7†). The accumulated images obtained with z-scan revealed that the **NRNO** probe can homogeneously stain the tissues, and that it is able to visualize NO at depths of up to 168 μm in mouse liver tissues under TP excitation.

**Fig. 5:**
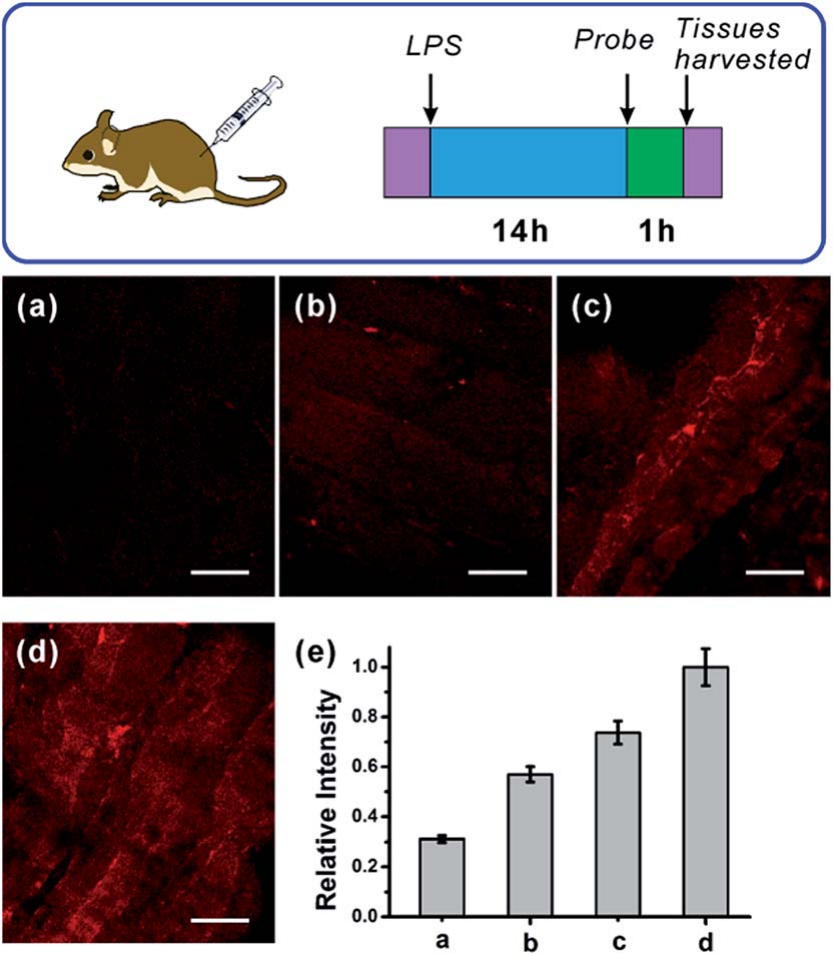
Detection of LPS-induced NO production using **NRNO**, in inflamed mouse tissues under TP microscope. (a–d) TP images of mice injected with various concentrations of LPS (0, 1.0, 2.0, 4.0 mg mL^–1^) followed by injection of 200 μL **NRNO** (200 μM). (e) Relative fluorescence intensities in (a–d). TP imaging depth for (a–d) was 100 μm. The TP excited fluorescence was collected at 620–700 nm upon excitation at 820 nm with a femtosecond pulse laser. Scale bar: 100 μm.

Immunology research has demonstrated that iNOS is highly expressed during infection and inflammation in the innate immune system, and high amounts of NO and other reactive nitrogen species are generated. These reactive nitrogen species exert toxic effects on invading bacteria or viruses *via* nitrosation and nitration of DNA, as well as oxidative inactivation of iron-sulfur centers, microbial proteins and cell wall components.^22^ It was also reported that LPS can cause inflammation in animals.^23^ However, so far there has been no report on monitoring NO generation in the progression of inflammation using a molecular probe. As the first trial, we utilized the **NRNO** probe to detect the NO production in a mouse model. To this end, 200 μL of LPS solutions with various concentrations (0, 1.0, 2.0, 4.0 mg mL^–1^) were separately injected subcutaneously into the left rear leg of four mice to cause inflammation, followed by the injection of **NRNO** at the same position. The sectioned tissue slices were then subjected to TP fluorescence imaging. Interestingly, the acquired high-resolution images present vivid visualizations of NO production in the immune response process. As illustrated in Fig. 5a, the tissue of the mouse injected with saline only (0 mg mL^–1^ LPS) showed negligible fluorescence, while the tissues from mice injected with LPS exhibited notable fluorescence enhancement (Fig. 5b–d). Furthermore, the fluorescence intensity of the tissues was positively correlated with the LPS concentration (Fig. 5e), which suggests that the NO generation is dependent on the dosage of the drug and could reflect the degree of inflammation. Taken together, the results confirm that the TP excited far-red emissive **NRNO** is sensitive enough for measuring NO levels in deep tissues, and could be a useful indicator in monitoring NO-related biological processes.

## Conclusions

In summary, we have developed a far-red emissive, two-photon **NRNO** probe, for nitric oxide and demonstrated its application in detecting NO in cells and tissues by two photon microscopy. The Nile Red-based fluorophore possesses both an adequate TP cross section and a far-red emission band centered at 650 nm, which are favourable for deep imaging in biological samples. The probe shows fast response, high sensitivity and specificity toward nitric oxide. **NRNO** is able to detect exogenous NO in HeLa cells and endogenously generated NO as stimulated by drugs in RAW 264.7 cells. It also exhibits high resolution in tissue imaging at depths up to *ca.* 170 μm. The probe is applicable in the monitoring of NO generation in the LPS-mediated inflammation of mice for the first time, which reveals that the NO concentration in inflamed tissue is positively correlated with the inflammation process. The results elucidate that the **NRNO** probe may afford a useful tool for studying the biological events involving NO. **NRNO** is the first far-red emissive two-photon probe for NO, which may also promote the advancement of TP fluorescence probes with long-wavelength emission.

## Supplementary Material

Supplementary information
