# Supplementary material for: NIR in, far-red out: developing a two-photon fluorescent probe for tracking nitric oxide in deep tissue
Source: Chem Sci. 2016 Apr 26;7(8):5230–5. doi: 10.1039/c6sc01313a (PMC6020528; doi:10.1039/c6sc01313a)
Supplement: Supplementary file 1 [file SC-007-C6SC01313A-s001.pdf]

Electronic Supplementary Material (ESI) for Chemical Science.
This journal is © The Royal Society of Chemistry 2016

Supporting Information

NIR in, Far-Red out: Developing a Two-Photon Fluorescent Probe for Tracking Nitric Oxide in Deep Tissue

Zhiqiang Mao, Wenqi Feng, Zhen Li, Lingyu Zeng, Weijie Lv, Zhihong Liu*

Key Laboratory of Analytical Chemistry for Biology and Medicine (Ministry of Education), College of Chemistry and Molecular Sciences, Wuhan University, Wuhan 430072, China.

Table of Content

1. Materials and apparatus……………………………………………………………………..S3
2. Experimental detail…………………………………………………………………………S4-S6
3. Synthesis and Characterization……………………………………………………..……S7-S9
4. Spectroscopic properties of NRNO……………………………………………..……….S10-S12
5. Cytotoxicity assay……………………………………………………………………………..S13
6. TPM images of NRNO stained liver tissues under Z-scan model……………………………….S14
7. NMR and HR-MS data……………………………………………………….................S15-S23
8. Reference……………………………………………………………………………………S24

1. Materials and Apparatus

Lipopolysaccharide (LPS), Interferon-γ (IFN-γ) and 6-(2-Hydroxy-1-methyl-2-nitrosohydrazino)-N-methyl-1-hexanamine (NOC-9, NO release: $t_{1/2}$ = 3 min (phosphate buffer at 22 °C)) were purchased from Sigma-Aldrich. 3-(4, 5-Dimethyl-2-thiazolyl)-2, 5-diphenyl-2H-tetrazolium bromide (MTT) and Hoechst 33258 were purchased from Aladdin Reagent, Co., Ltd (Shanghai, China). Other regents were from commercial sources and used without further purification. All reactions were performed under argon atmosphere unless otherwise stated. Anhydrous solvents for organic synthesis were prepared by standard methods. All aqueous solutions were prepared in ultrapure water with a resistivity of 18.25 MΩ•cm (purified by the Milli-Q system supplied by Millipore). Two-photon excited fluorescence data were measured by exciting with a mode-locked Ti: sapphire femtosecond pulsed laser (Chameleon Ultra I, Coherent Inc.) with a pulse width of 140 fs and repetition rate of 80 MHz. The two-photon excited fluorescence intensity was recorded on a DCS200PC Photon Counting (Beijing Zolix Instruments Co., Ltd.) with single-photon sensitivity through an Omni-λ5008 monochromator (Beijing Zolix Instruments Co., Ltd.). One-photon excited fluorescence was measured on a RF-5301 fluorescence spectrophotometer (Shimadzu Scientific Instruments Inc.). Absorption measurements were conducted on a UV2550 UV-vis spectrophotometer (Shimadzu Scientific Instruments Inc.). Two-photon microscopy was performed on a Zeiss Axio Examiner LSM 780 multiphoton laser scanning confocal microscope (Carl Zeiss, Germany).

2. *Experimental detail*

Spectroscopic Measurements The fluorescence quantum yield was determined with Nile Red in methanol (Φ=0.38) as the reference with a literature method. [1] Nitric oxide (NO) stock solution (1.9 mM) was prepared by purging PBS (0.01 M, pH 7.4) with N_2 gas for 30 min and then with NO gas (99.5 %) for 30 min at 25 °C. Other ROS and RNS were prepared according to previous literature. [2]

Measurement of Two-photon Cross Section The two-photon cross section (δ) was determined by using femtosecond (fs) fluorescence measurement technique as described.[3] Probe (5.0×10^{-6} M) was dissolved in 10 mM PBS buffer (pH 7.4, containing 10% DMF), and the two-photon induced fluorescence intensity was measured at 750-900 nm by using rhodamine B as the reference, whose two-photon property has been well characterized in the literature. The intensities of the two-photon induced fluorescence spectra of the reference and sample at the same excitation wavelength were determined. The 2P absorption cross section was calculated by using $\delta=\delta_r[S_s\Phi_r\phi_r c_r]/(S_r\Phi_s\phi_s c_s)$, where the subscripts s and r stand for the sample and reference molecules. The intensity of the two-photon excited fluorescence was denoted as S. Φ is the fluorescence quantum yield, and ϕ is the overall fluorescence collection efficiency of the experimental apparatus. The number density of the molecules in solution was denoted as c. δ_r is the 2P absorption cross section of the reference molecule.

Experimental Calculation of Limit of Detection (LOD) Value The detection limit was calculated based on the method reported in the previous literature.[4] The fluorescence emission spectrum of **NRNO** was measured by eleven times and the standard deviation of blank measurement was achieved. The fluorescence intensity at 650 nm was plotted as a concentration of NO. The detection limit was calculated by using detection limit $3\sigma/m$: where σ is the standard deviation of blank measurement, m is the slope between the fluorescence intensity versus NO concentration.

Cytotoxicity Assay The cytotoxicity was evaluated by MTT assay. HeLa cells were cultured in Dulbecco's modified Eagle's medium (DMEM) in 96-well microplates at 37 °C under 5% CO_2 for 12 h. The medium was next replaced by fresh medium containing various concentrations of NRNO (0-30 μM/mL). Each concentration was tested in three replicates. Cells were rinsed twice with phosphate buffer saline 24 h later and incubated with 0.5 mg/mL MTT reagent for 4h at 37 °C. 100 μL of DMSO was then added to dissolve formazan. The absorbance at 490 nm was measured in a microplate reader. Cell viability (%) was calculated according to following equation: Viability = (mean Abs. of treated wells/mean Abs. of control wells) ×100%.

Cell Culture and Imaging HeLa cells were cultured with DMEM supplemented with 10% (v/v) newborn calf serum (Gibco), 100 U•mL^{-1} penicillin, and 100 μg•mL^{-1} streptomycin in a humidified atmosphere with 5/95 (v/v) of CO_2/air at 37 °C. One day before imaging, cells were detached with a treatment of 0.2% (w/v) trypsin-EDTA solution (Gibco) and suspended in culture media. The cell suspension was then transferred to confocal dishes to grow with adherence. For probe loading, the growth medium was replaced with 5.0 μM **NRNO** and 1.0 μM Hochest 33258 in culture media and incubated at 37 °C under 5% CO_2 for 30 min. Next, the cells were washed with serum-free DMEM for three times. Various concentration of NOC-9 solution was added to the dishes and incubated at 37 °C under 5% CO_2 for 30 min. Raw 264.7 cells were maintained with DMEM supplemented with 10% (v/v) newborn calf serum (Gibco), 100 U•mL^{-1} penicillin, and 100 μg•mL^{-1} streptomycin in a humidified atmosphere with 5/95 (v/v) of CO_2/air at 37 °C. For confocal imaging, RAW 264.7 cells at 80% confluence were harvested by scraping and transferred to confocal dishes to grow with adherence. For endogenous NO production, RAW 264.7 cells were pretreated with 20 μg/mL LPS，200 U/mL IFN-γ and 0.5 mg/mL L-arginine for 14 hours. Then, Raw 264.7 cells were incubated with 5.0 μM **NRNO** at 37 °C for 30 min and washed with serum-free DMEM three times for imaging. One-photon and two-photon excited fluorescence images were obtained by Zeiss Axio Examiner LSM 780 multiphoton laser scanning confocal microscope.

Tissue Imaging After the Kunming mice (~35 g) anesthesia, 200 μL of LPS solution with various concentrations (0-4.0 mg/mL) was subcutaneously injected on left rear leg of the mice. After 14h, 200 μL of 200 μM **NRNO** was intramuscularly injected to the LPS-mediated inflammation area. 1 h later, the mice were anesthetized, the skin of the inflamed tissues were harvested and embedded in tissue-freezing medium, frozen and consecutively sectioned into slices. Then, the slices were washed with PBS three times and imaged by two-photon microscope. Animal care and handing procedures were reviewed and approved by Animal Care and Use Committee of Wuhan University.

3. *Synthesis and Characterization*

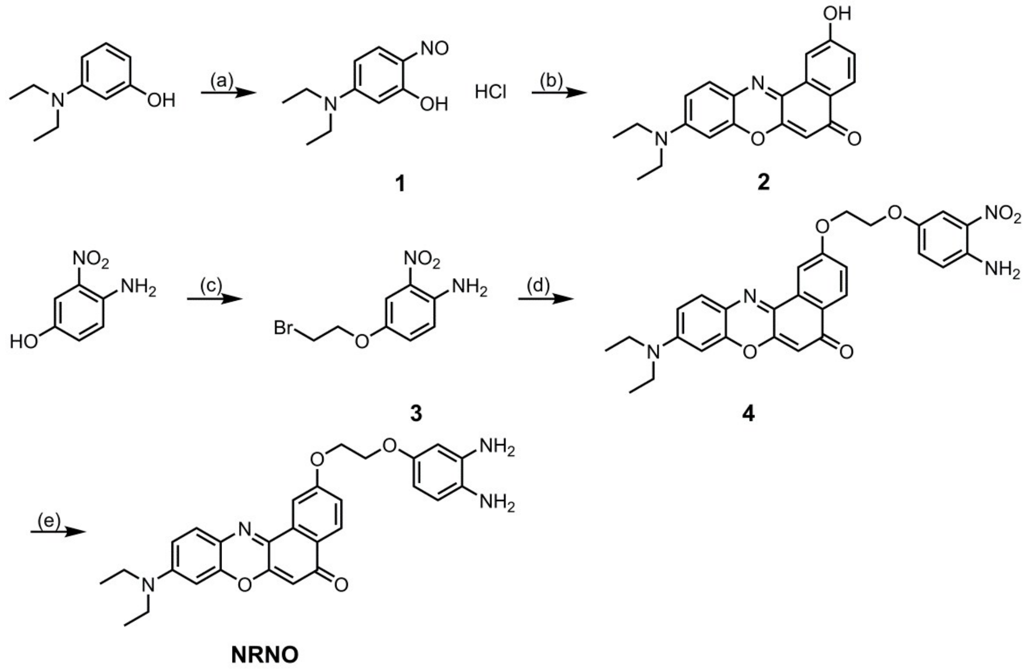

Fig. S1 Structure and synthesis of **NRNO**. Reagents and conditions: (a) $NaNO_2$, 37% HCl, H_2O, 0-5°C; (b) 1, 6-dihydroxynaphthalene, DMF, 140°C; (c) 1, 2-dibromoethane，K_2CO_3, CH_3CN, 85°C; (d) Compound 2, K_2CO_3, KI, TBAI, DMF, 80°C; (e) 5% Pd/C, 85%N_2H_4 •H_2O, THF, reflux.

Synthesis of Compound 1. 3.36 g (20 mmol) of 3-diethylaminophenol was dissolved in 8.0 mL of concentrated HCl and 20 g of ice. A cold solution of 1.68 g (24 mmol) of $NaNO_2$ in 6 mL of deionized water was added dropwise to the above solution (during ca. 1h) and the temperature was kept at 0-5 °C. The resulting slurry was stirred for another 2h at 0 °C. The resulting precipitate was filtered and washed with 10 mL of 4M HCl solution, dried to yield yellow powder (3.10g, 67%). The crude product was pure enough from ^1H NMR analysis and used for next reaction without further purification. ^1H NMR (400 MHz, DMSO-d_6) δ 7.57 (d, J = 10.4 Hz, 1H), 7.23 (m, 1H), 6.65 (d, J = 1.9 Hz, 1H), 3.83 (m, 4H), 1.28 (t, J = 6.9 Hz, 6H).

Synthesis of Compound 2. 0.80 g (5.0 mmol) of 1, 6-dihydroxy naphthalene was added to a solution of 2-nitrosophenol (1.15g, 5.0 mmol) in 20 mL of DMF under argon atmosphere. The mixture was reflux at 140 °C for 5h. After the mixture cooled down to room temperature, 100 mL of water was added to the mixture and extracted with dichloromethane, dried over anhydrous Na_2SO_4. The solvent was removed and the product was purified by silica gel column chromatography (ethyl acetate: dichloromethane, 1:10) to yield dark green solid (0.42g, 25%). ^1H NMR (400 MHz, DMSO-d_6) δ 10.45 (s, 1H), 7.97 (d, J = 8.6 Hz, 1H), 7.88 (d, J = 2.4 Hz, 1H), 7.57 (d, J = 9.1 Hz, 1H), 7.09 (dd, J = 8.6, 2.5 Hz, 1H), 6.80 (d, J = 2.6 Hz, 1H), 6.63 (d, J = 2.5 Hz, 1H), 6.15 (s, 1H), 3.49 (q, J = 7.0 Hz, 4H), 1.16 (t, J = 7.0 Hz, 6H).

Synthesis of Compound 3. 852 μL (10.0 mmol) of 1, 2-dibromoethane was added to a mixture of 308 mg (2.0 mmol) of 4-amino-3-nitrophenol and 544 mg (4 mmol) of K_2CO_3 in 6.0 mL of CH_3CN. The mixture was refluxed overnight at 85 °C under argon atmosphere. The mixture was filtrated and the filtrate was evaporated to dryness. The crude product was purified by silica gel chromatography (ethyl acetate: petroleum ether, 1:6) to yield red powder (428 mg, 82%). ^1H NMR (400 MHz, DMSO-d_6) δ 7.41 (d, J = 2.9 Hz, 1H), 7.32 (s, 2H), 7.22 (dd, J = 9.2, 2.9 Hz, 1H), 7.02 (d, J = 9.2 Hz, 1H), 4.35-4.24 (m, 2H), 3.84-3.74 (m, 2H). ^{13}C NMR (100 MHz, DMSO-d_6) δ 148.05, 142.69, 129.39, 127.96, 121.29, 107.12, 68.90, 31.86. HRMS (Direct Analysis in Real Time (DART)) Calcd. for $C_8H_{10}O_3N_2Br$ $[M+H]^+$: 260.9869. Found 260.9869.

Synthesis of Compound 4. To a solution containing 200 mg (0.60 mmol) of compound **2** and 234 mg (0.90 mmol) of compound **3** in 9 mL of DMF was added 166 mg (1.2 mmol) of K_2CO_3, 100 mg (0.60 mmol) of KI and 44 mg (0.12 mmol) of tetrabutylammonium iodide (TBAI). The mixture was stirred at 80 °C overnight under argon atmosphere. After the reaction completed, 50 mL of water was added to the mixture and extracted with 200 mL of ethyl acetate for three times, dried over anhydrous Na_2SO_4. After the removal of solvent, the product was purified by silica gel column chromatography (ethyl acetate: dichloromethane, 1:10) to yield dark green solid (126 mg, 41%). ^{1}H NMR (400 MHz, DMSO-*d*$_6$) δ 8.06 (d, *J* = 8.7 Hz, 1H), 8.00 (s, 1H), 7.62 (d, *J* = 9.0 Hz, 1H), 7.50 (d, *J* = 2.7 Hz, 1H), 7.46-7.19 (m, 4H), 7.03 (d, *J* = 9.2 Hz, 1H), 6.83 (d, *J* = 8.9 Hz, 1H), 6.66 (s, 1H), 6.20 (s, 1H), 4.51 (s, 2H), 4.38 (s, 2H), 3.51 (q, *J* = 6.7 Hz, 4H), 1.18 (t, *J* = 6.7 Hz, 6H). HRMS (DART) Calcd. for $C_{28}H_{27}O_6N_4$ $[M+H]^+$: 515.1925. Found 515.1924.

Synthesis of NRNO. To a solution containing 50 mg (0.1 mmol) of compound **4** and 0.5 mL of N_2H_4•H_2O in 20 mL of THF, 50 mg of 5% Pd/C was added to the above solution and refluxed for 1.5 h. The mixture was filtrated, evaporated to dryness and re-dissolved in 100 mL of dichloromethane. Then the solution was washed with saturated NaCl solution twice and saturated $NaHCO_3$ solution once, dried over anhydrous Na_2SO_4 and evaporated to dryness to get dark green powder (25mg, 47%). ^{1}H NMR (400 MHz, DMSO-*d*$_6$) δ 8.05 (d, *J* = 8.6 Hz, 1H), 7.98 (s, 1H), 7.62 (d, *J* = 8.7 Hz, 1H), 7.31 (d, *J* = 8.2 Hz, 1H), 6.82 (d, *J* = 8.7 Hz, 1H), 6.65 (s, 1H), 6.45 (d, *J* = 8.1 Hz, 1H), 6.25 (s, 1H), 6.20 (s, 1H), 6.08 (d, *J* = 7.5 Hz, 1H), 4.32 (m, 8H), 3.51 (d, *J* = 6.6 Hz, 4H), 1.18 (t, *J* = 6.5 Hz, 6H). ^{13}C NMR (150 MHz, DMSO-*d*$_6$) δ 181.87, 161.50, 152.22, 151.53, 151.30, 146.93, 138.61, 137.11, 134.04, 131.45, 129.19, 127.74, 125.48, 124.41, 118.55, 115.70, 110.57, 106.72, 104.51, 102.90, 102.48, 96.41. 67.55, 66.78, 46.02, 12.93. HRMS (DART) Calcd. for $C_{28}H_{29}O_4N_4$ $[M+H]^+$: 485.2183. Found 485.2181.

4. Spectroscopic properties of NRNO

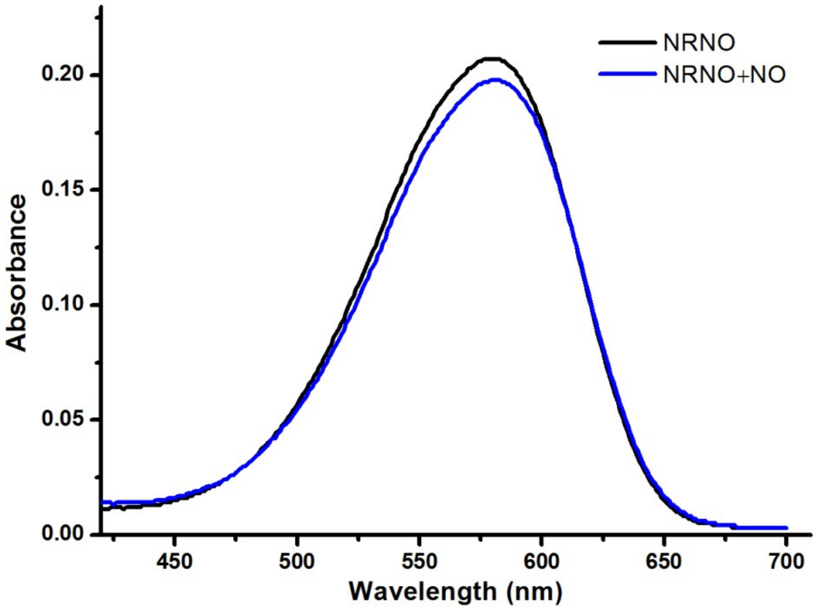

Fig. S2 Absorption spectra of NRNO before and after reacting with excess NO. These data were measured in 10 mM PBS (pH=7.4， containing 10% DMF as co-solvent).

Table S1. Photophysical data for **NRNO** before and after reacting with excess NO in buffer solution.

Compd[a]	λ_{max}^{1}[b]	ε[c]	λ_{max}^{1}[d]	Φ[e]	$\lambda_{max}^{(2)}$[f]	$\Phi\delta$[g]
**NRNO**	583	4.00×10^{4}	648	0.03	nd[h]	nd[h]
**NRNO+NO**	585	3.96×10^{4}	650	0.42	820	38

[a] All data were measured in 10 mM PBS (pH=7.4, containing 10% DMF as co-solvent) in the absence and presence of 30 μM NO. [b, d] λ_{max} of the absorption and one-photon emission spectra in nm. [c] Fluorescence quantum yield.[5] [f] λ_{max} of the two-photon emission spectra in nm. [g] Two-photon action cross section in GM.[6] [h] The two-photon excited fluorescence intensity was too weak to measure the two-photon action cross section accurately.

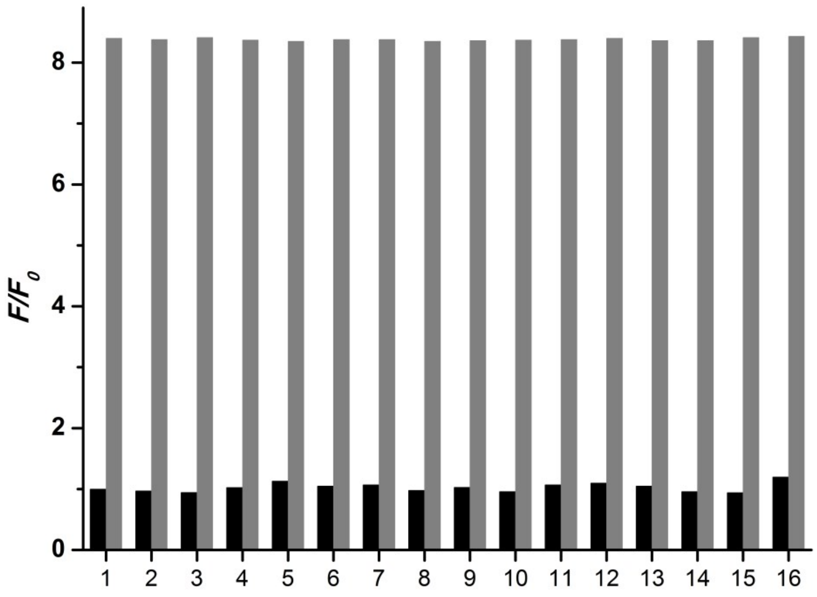

Fig. S3 Fluorescence enhancement factor (*F/F_0*) of 5.0 μM **NRNO** toward 30 μM NO in the presence of various interfering species (grey bars). Black bars are signals with the interfering species only. 50 μM for metal ions (2-6), 1 mM for biothiols (7-9), 30 μM for ROS/RNS (10-16): (1) blank, (2) Ca^{2+}, (3) Mg^{2+}, (4) Zn^{2+}, (5)Mn^{2+}, (6) Fe^{2+}, (7) GSH, (8) Cys, (9) Hcy, (10) •OH, (11) $^1O_2$, (12) H_2O_2, (13) ClO^-, (14) O_2^-, (15) NO_2^-, (16) $ONOO^-$. The excitation wavelength was 585 nm, and the fluorescence intensity was measured at 650 nm.

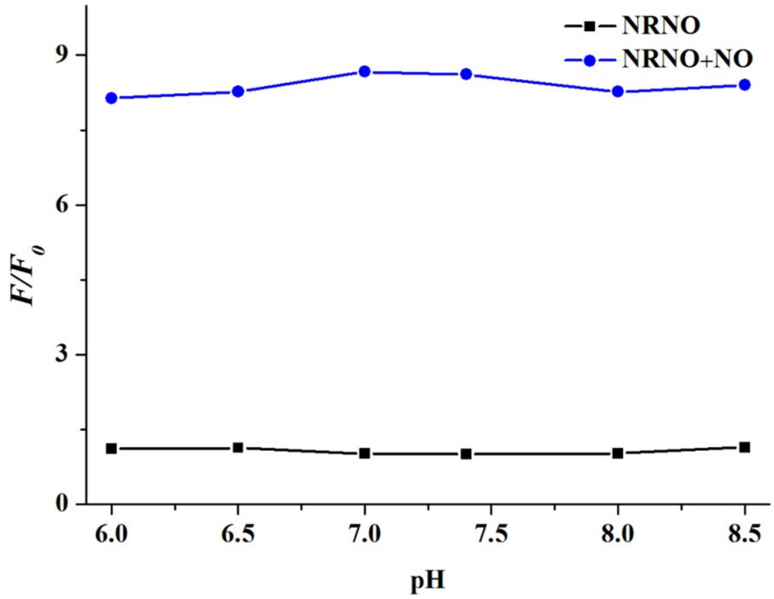

Fig. S4 Effect of pH on the fluorescence of 5.0 μM **NRNO** before and after reacting with 30 μM NO. The excitation wavelength was 585 nm, and the fluorescence intensity was measured at 650 nm in 10 mM PBS (pH = 7.4, containing 10% DMF as co-solvent).

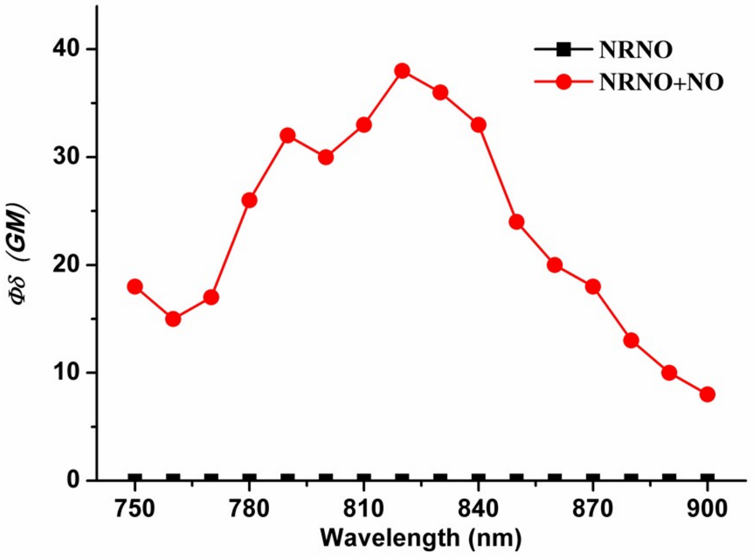

Fig. S5 Two-photon excitation spectra of 5.0 μM **NRNO** in the presence or absence of 30 μM NO in 10 mM PBS (pH=7.4, containing 10% DMF as co-solvent).

5. *Cytotoxicity Assay*

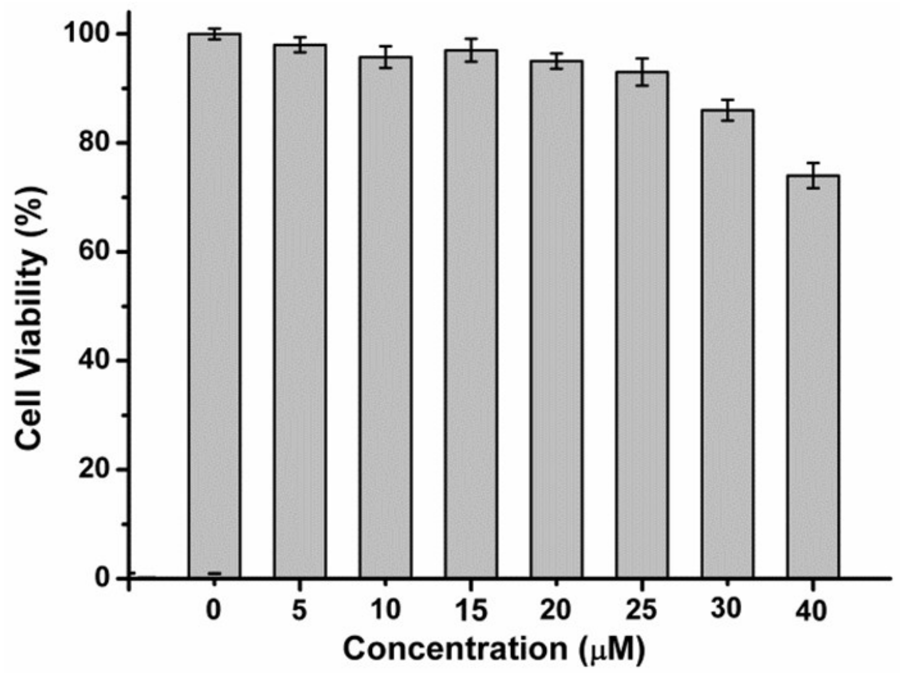

Fig. S6 Viability of HeLa cells incubated with various concentrations (0-40 μM) of **NRNO** for 24h as measured by MTT assay.

6. TPM Images of NRNO Stained Liver Tissues under Z-scan Model

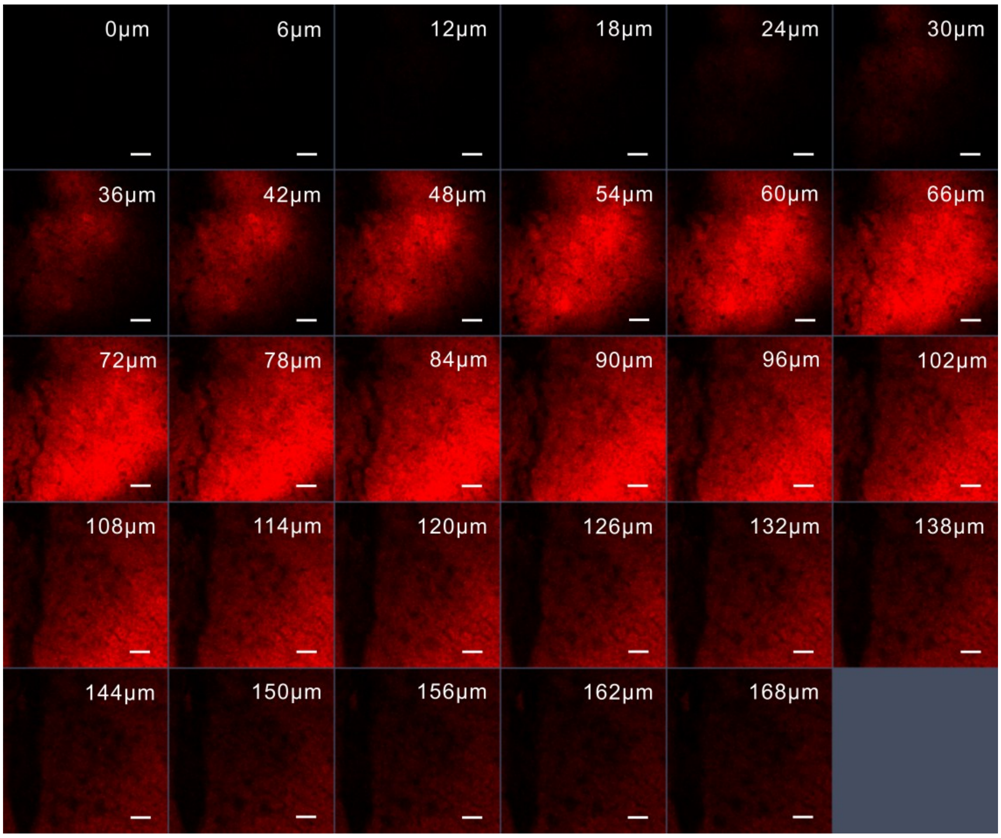

Fig. S7 Z-direction 2P images for **NRNO**-stained rat liver tissue incubated with 60 μM NO. The TP fluorescence was excited at 820 nm and collected at 600-700 nm. Scale bar: 50 μm.

7. NMR and HR-MS Data

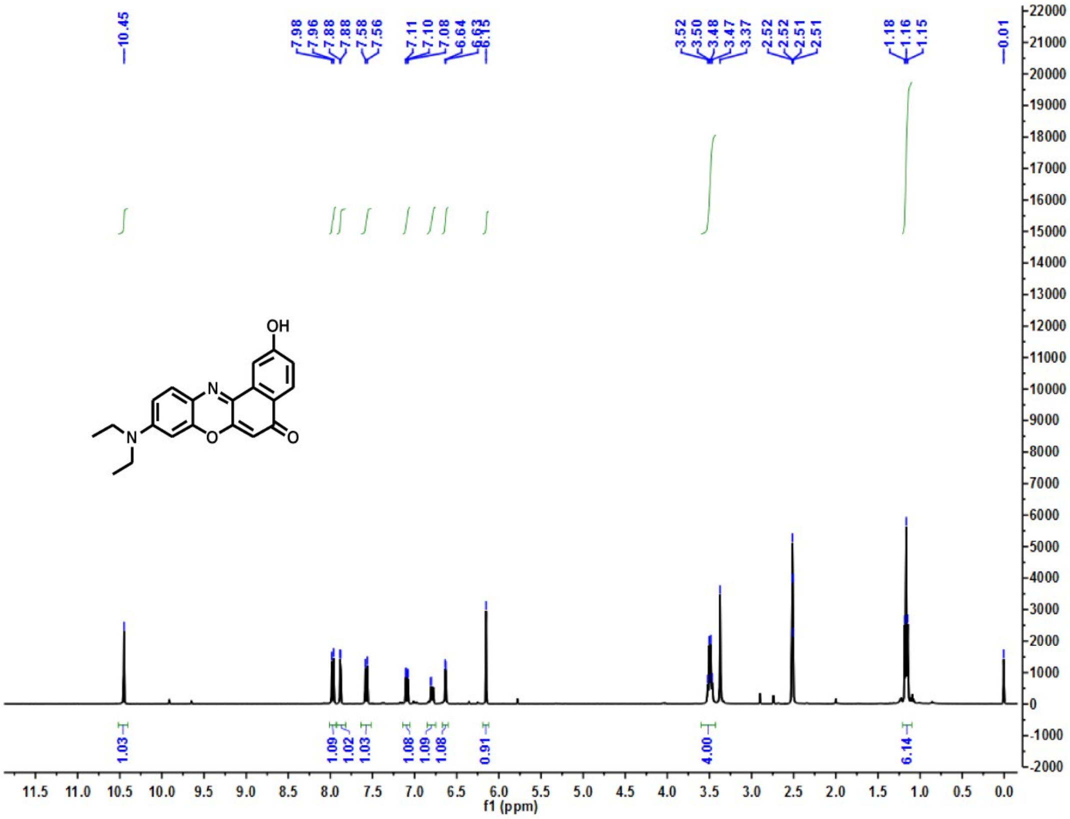

Fig. S8 ^1H NMR spectrum of compound **2** (DMSO-d_6, 298K, 400 MHz).

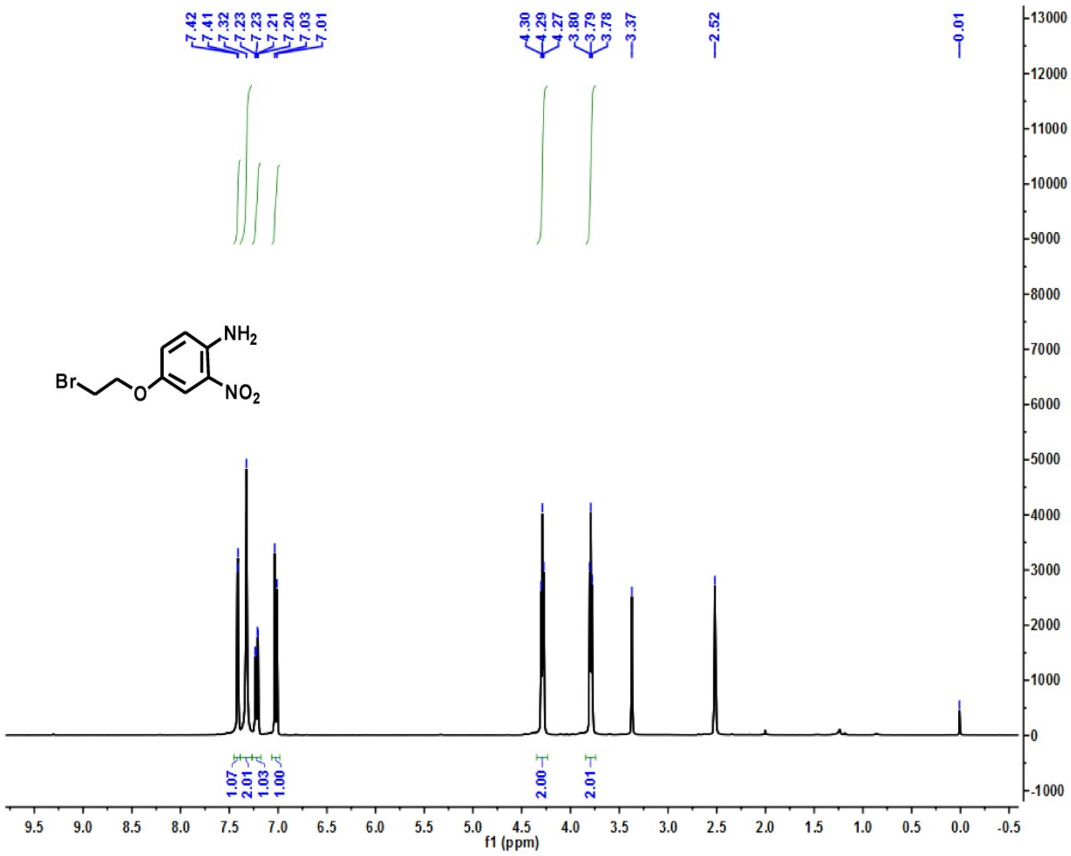

Fig. S9 ^{1}H NMR spectrum of compound **3** (DMSO-d_6, 298K, 400 MHz).

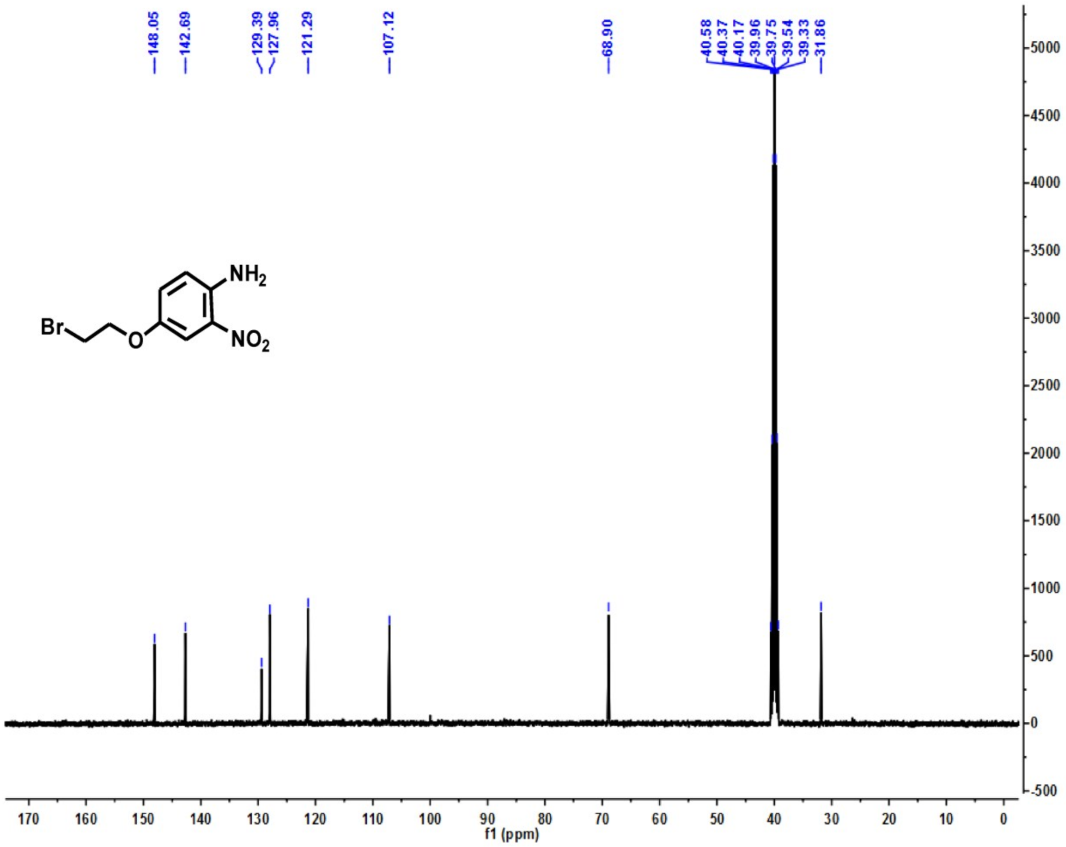

Fig. S10 ^{13}C NMR spectrum of compound **3** (DMSO-d_6, 298K, 100 MHz).

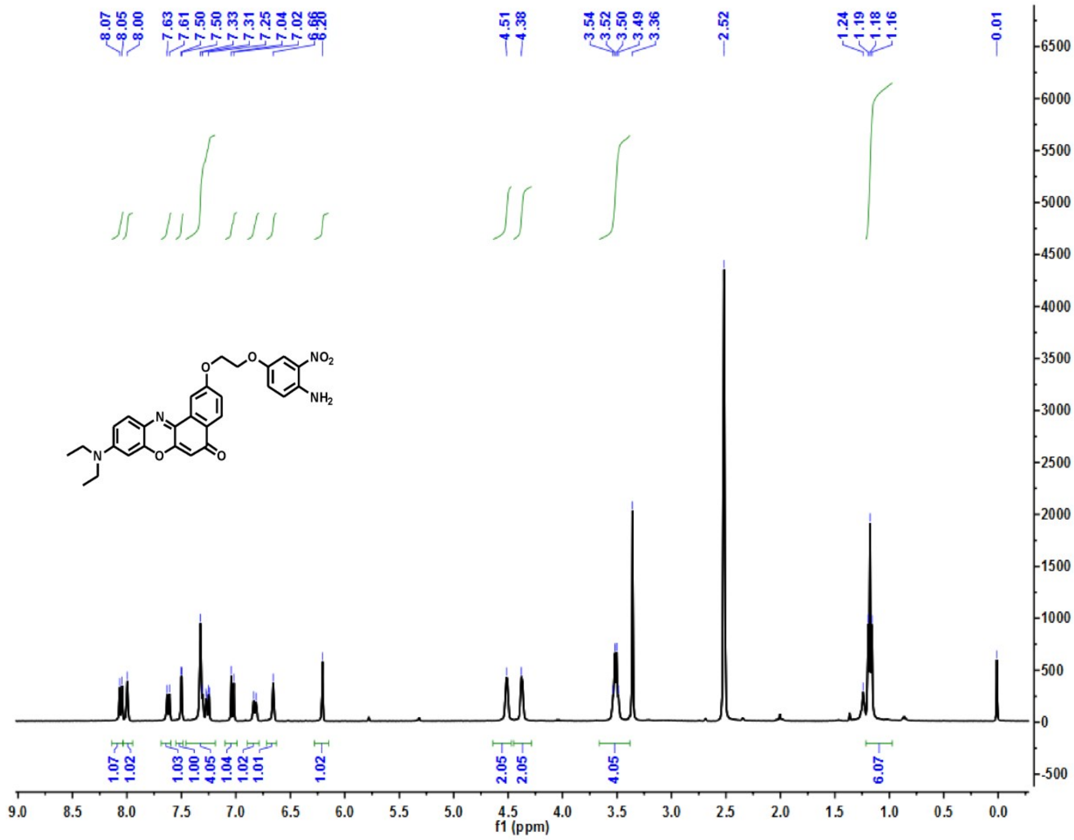

Fig. S11 ^{1}H NMR spectrum of compound **4** (DMSO-d_6, 298K, 400 MHz).

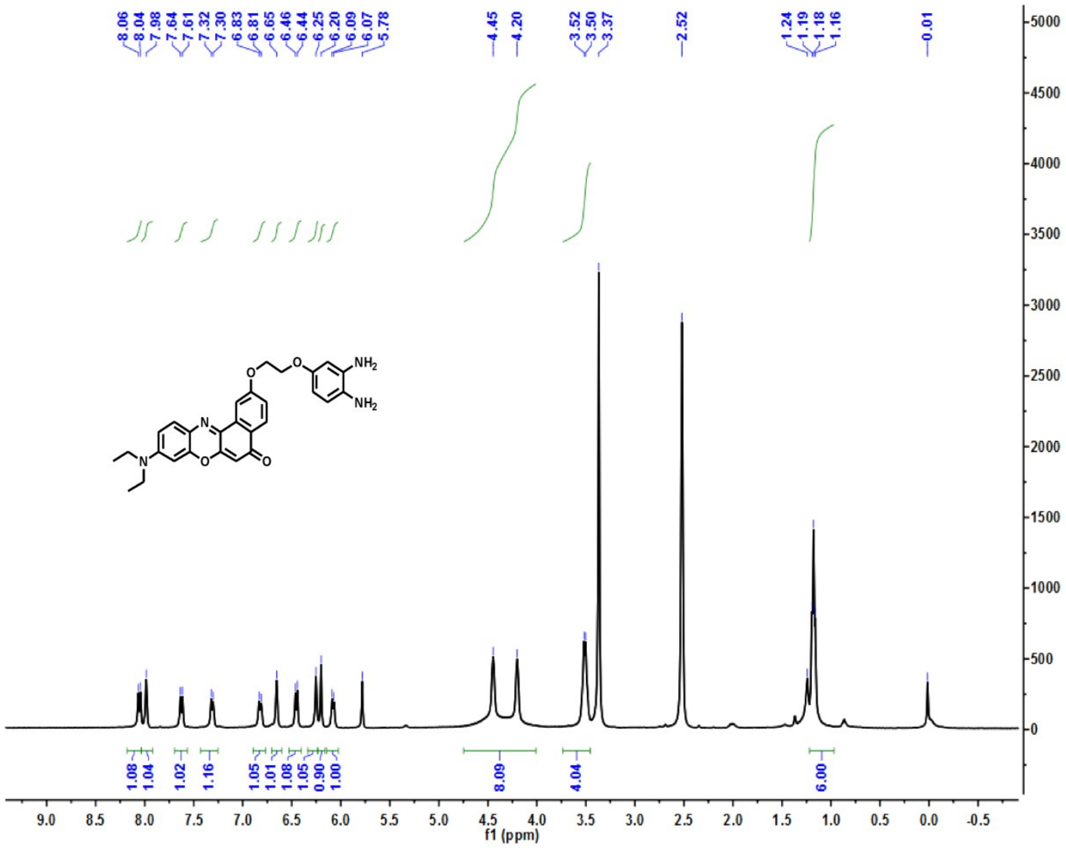

Fig. S12 ^{1}H NMR spectrum of compound **NRNO** (DMSO-d_6, 298K, 400 MHz).

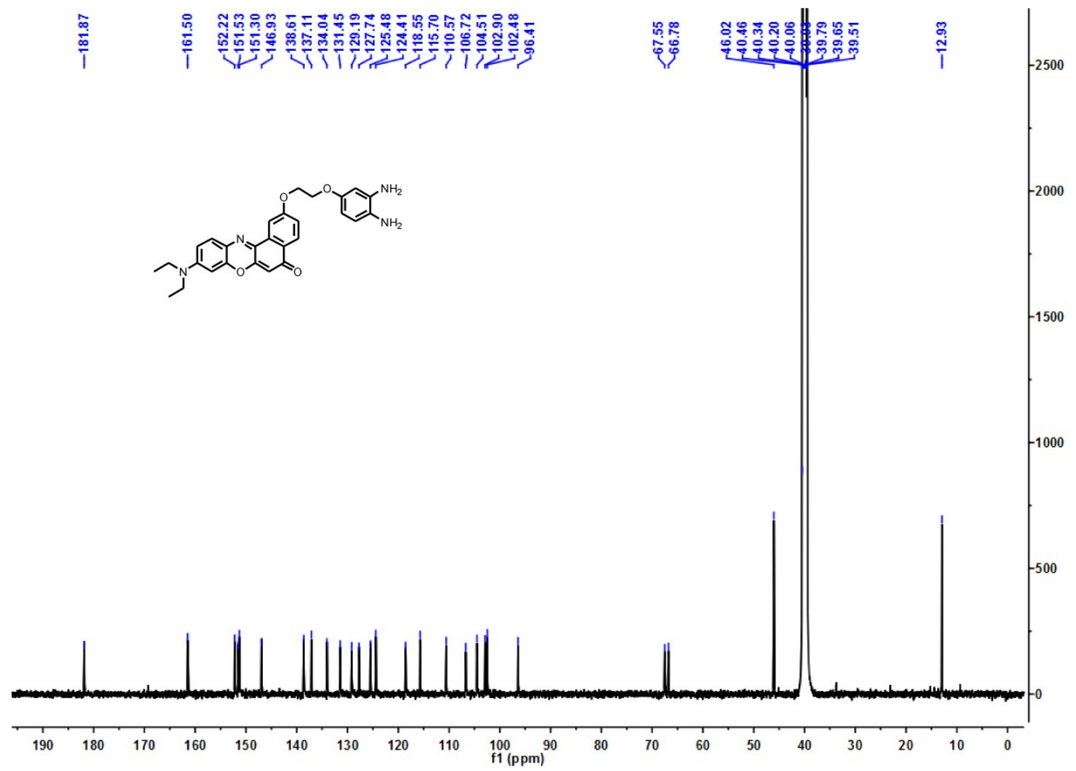

Fig. S13 ^{13}C NMR spectrum of the compound **NRNO** (DMSO-d_6, 298K, 150 MHz).

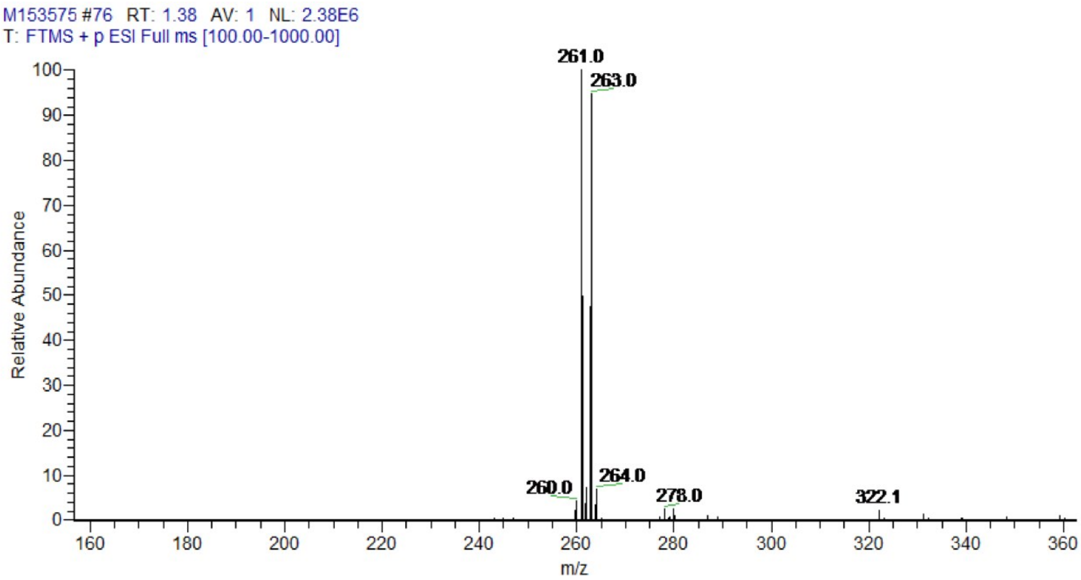

Elemental composition search on mass 260.99

m/z= 255.99-265.99

m/z	Theo. Mass	Delta (ppm)	RDB equiv.	Composition
260.9869	260.9869	-0.08	4.5	$C_8 H_{10} O_3 N_2 Br$

Fig. S14 HRMS spectrum of compound **3.**

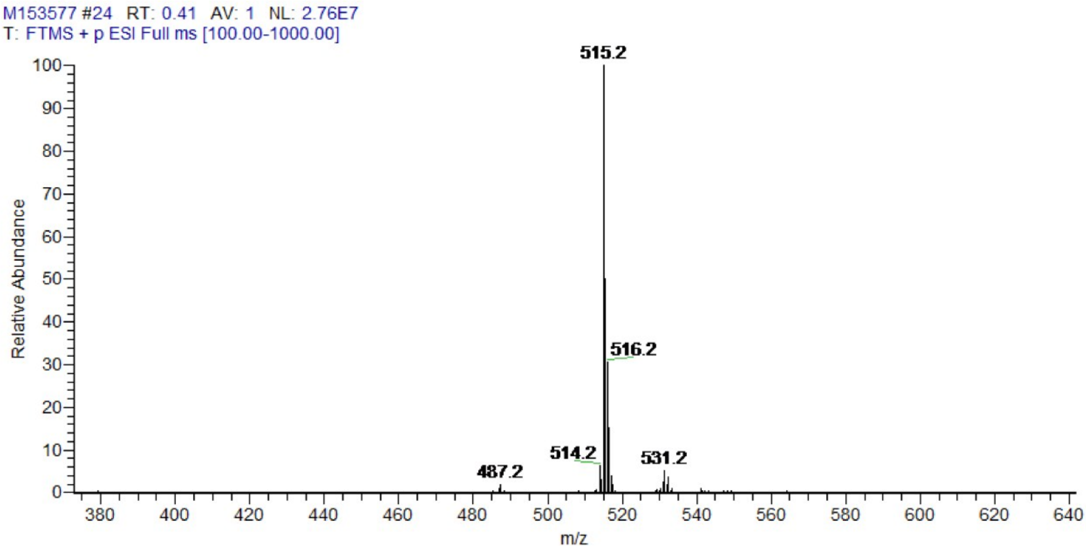

Elemental composition search on mass 515.19

m/z= 510.19-520.19

m/z	Theo. Mass	Delta (ppm)	RDB equiv.	Composition
515.1924	515.1925	-0.14	17.5	$C_{28}H_{27}O_{6}N_{4}$
	515.1912	2.46	12.5	$C_{27}H_{31}O_{10}$
	515.1939	-2.74	17.0	$C_{30}H_{29}O_{7}N$

Fig. S15 HRMS spectrum of compound **4.**

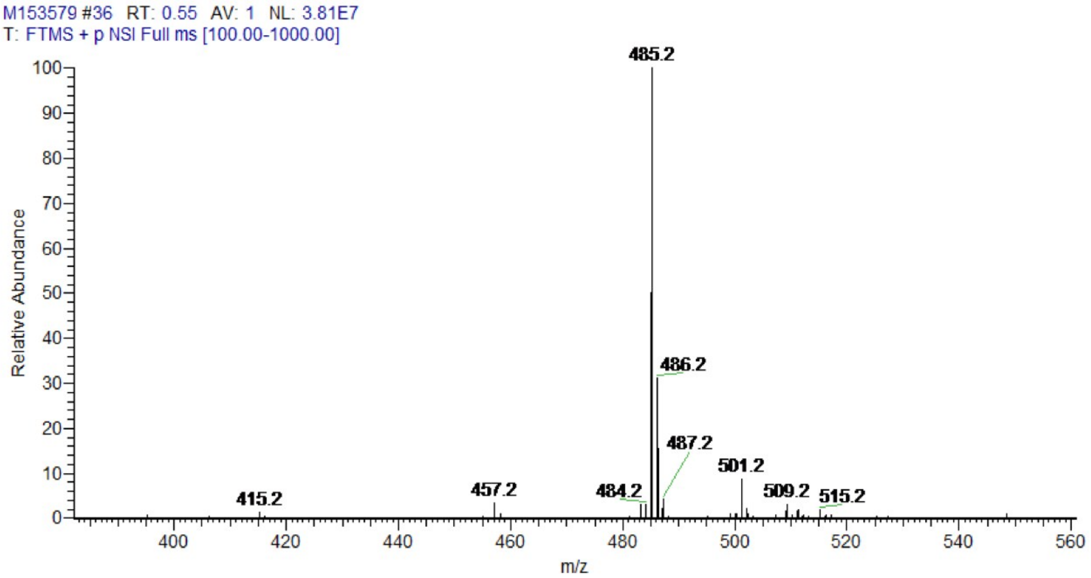

Elemental composition search on mass 485.22

m/z= 480.22-490.22

m/z	Theo. Mass	Delta (ppm)	RDB equiv.	Composition
485.2181	485.2183	-0.48	16.5	$C_{28}H_{29}O_4N_4$
	485.2170	2.28	11.5	$C_{27}H_{33}O_8$
	485.2197	-3.24	16.0	$C_{30}H_{31}O_5N$

Fig. S16 HRMS spectrum of compound **NRNO.**

8. Reference

(1) O. A. Kucherak, S. Oncul, Z. Darwich, D. A. Yushchenko, Y. Arntz, P. Didier, Y. Mély, A. S. Klymchenko, *J. Am. Chem. Soc.* ***2010***, *132*, 4907.

(2) L. Yuan, L. Wang, B. K. Agrawalla, S. J. Park, H. Zhu, B. Sivaraman, J. Peng, Q. H. Xu, Y. T. Chang, *J. Am. Chem. Soc.* **2015**, *137*, 5930.

(3) C. Xu, W. W. Webb, *J. Opt. Soc. Am. B* **1996**, *13*, 481.

(4) B. Zhu, C. Gao, Y. Zhao, C. Liu, Y. Li, Q. Wei, Z. Ma, B. Du, X. Zhang, *Chem. Commun.* **2011**, 47, 8656-8658.bu

(5) J. N. Demas, G. A. Crosby, *J. Phys. Chem.* **1971**, *75*, 991.

(6) S. K. Bae, C. H. Heo, D. J. Choi, D. Sen, E. H. Joe, B. R. Cho, H. M. Kim, *J. Am. Chem. Soc.* **2013**, *135*, 9915.
